# Temporal incidence and impact of dementia in rheumatoid arthritis: a cohort study

**DOI:** 10.1016/j.ero.2026.03.007

**Published:** 2026-04-08

**Authors:** Angela Marie Chan, Renee Ng, Charles Inderjeeth, Johannes Nossent

**Affiliations:** 1 Department of General Medicine, Geronto-Rheumatology, Khoo Teck Puat Hospital, Singapore, Singapore; 2 Rheumatology Group, School of Medicine, University of Western Australia, Crawley, WA, Australia; 3 Department of Rehabilitation and Aged Care, Sir Charles Gairdner Hospital, Perth, WA, Australia

## Abstract

**Objectives:**

The objective of this study is to address conflicting evidence that chronic inflammation may increase the risk of dementia in patients with rheumatoid arthritis (RA).

**Methods:**

Retrospective population-based study using longitudinally linked administrative health data over a 30-year period for ever hospitalised patients with RA (n = 14,041, age 64 years, 67.2% female) and controls (n = 33,785, age 65 years, 65.6% female). Dementia was defined by the International Classification of Diseases codes for Alzheimer’s disease (AD), vascular dementia, and nonspecific dementia subtypes. Dementia incidence rate (IR) and mortality rate (MR) per 1000 person-years and comorbidities are reported.

**Results:**

During 9.6 years of follow-up, 1463 (10.4%) of patients with RA and 3701 (11%) of controls were diagnosed with dementia at respective age of 83 vs 84 years (*P* = .01). The IR was 12.07 (95% CI: 11.15-12.71) in patients with RA and 11.59 (95% CI: 11.22-11.97) in controls corresponding to an IR ratio of 1.04 (95% CI: 0.98-1.11, *P* = .21), which did not change significantly over 3 decades. Traditional risk factors for dementia were equal in both groups, but patients with RA with dementia were less likely to be classified as AD (odds ratio = 0.59, 95% CI: 0.48-0.73, *P* < .001). Hospitalisation rates after dementia diagnosis were higher for patients with RA, and the crude MR (overall 91.8 vs 90.4, *P* = .64) remained similar before or after 2000 for both groups.

**Conclusions:**

There was no difference in temporal incidence and MRs for dementia between patients with RA and matched controls. These data suggest that there is minimal impact of RA on the frequency and outcome of dementia.

**WHAT IS ALREADY KNOWN ON THIS TOPIC**

• Chronic inflammation is considered an additional risk factor for dementia.
• There is inconsistent evidence about the role of rheumatoid arthritis (RA) as a risk factor for dementia and its subtypes.

**WHAT THIS STUDY ADDS**

• Over a 30-year period, the incidence of dementia did not differ for patients with RA and matched controls.
• Patients with RA were less likely to be diagnosed with Alzheimer’s disease (AD) but had similar rates of vascular dementia as controls.
• Mortality rates for patients with RA with dementia were similar to those of controls with dementia.

**HOW THIS STUDY MIGHT AFFECT RESEARCH, PRACTICE OR POLICY**

• RA cannot be considered an independent risk factor for dementia.
• The introduction of more aggressive synthetic-biological disease-modifying antirheumatic drug treatment in RA did not reduce the proportion of patients with RA diagnosed with AD over the first 10 years, but this may change with more prolonged follow-up.

Alt-text: Unlabelled box dummy alt text

## INTRODUCTION

Chronic systemic inflammation is a hallmark of rheumatoid arthritis (RA), and while inflammation has been associated with dementia [1], especially Alzheimer’s disease (AD), studies on the association between RA and dementia/AD have had mixed results [2]. The presence of self-reported midlife RA in a Swedish population study was associated with a 40% increase in the risk of cognitive decline and incident dementia later in life [3]. The presence of cardiovascular risk factors and events, as well as rheumatoid nodules, is associated with an elevated risk of incident dementia among patients with RA in the Rochester Epidemiology Project (REP) [4]. However, erosive and seropositive RA did not increase the risk of dementia in REP, and no distinction was made between the subtypes of dementia. Although these studies provide some support that chronic inflammation increases the risk of dementia, a meta-analysis found that the risk for dementia in systemic rheumatic diseases was elevated for patients with osteoarthritis, systemic lupus erythematosus, and Sjogren’s disease, but not for participants with RA, with a pooled risk ratio of 0.98 (95% CI: 0.90-1.07) [5]. Nearly half a million Australians are living with dementia, and this number is expected to more than double by 2058 [6,7]. Given the uncertainties as to whether RA is a risk factor for dementia, this study compared the frequency, risk factors, and outcomes of dementia in patients with RA and age- and sex-matched controls.

## METHODS

### Study design

A retrospective population-based study using prospectively collected state-wide linked administrative health data was conducted.

### Data sources

The Western Australian Rheumatic Disease Epidemiological Registry (WARDER) contains deidentified longitudinally linked health data for all hospital-ascertained patients with inflammatory rheumatic disease in the Western Australia (WA) Hospital Morbidity Data System (HMDS), Emergency Department Data Collection, and the WA cancer and death registries. All registered data are based on the most appropriate clinical diagnoses in discharge notes by treating physicians at the end of hospital visits. In WA, the physician discharge notes are then verified by professionally trained clinical coders to ensure uniform translation into the International Classification of Diseases (ICD) codes following current Australian Classification pathways. This elaborate process avoids direct coding by healthcare providers who are less familiar with correct ICD coding and avoids billing for profitability. WARDER data were linked by the WA Data Linkage Branch through probabilistic matching and clerical review, with high linkage accuracy, and provide individual longitudinal health records for each participant [8]. WARDER has been successfully applied earlier for the study of various rheumatic diseases [9,10].

### Participants

Patients with RA aged 16 years and older admitted at least once to a public or private hospital in WA from January 1, 1985 to December 31, 2014 were identified by primary or secondary ICD codes for RA (ICD, 9th Revision, Australian Modification 714.00-714.99 and ICD, 10th Revision, Australian Modification [10-AM] M05.00-M06.99). To increase accuracy, patients with RA were excluded if they had at least 2 subsequent health contacts identifying other forms of arthritis, including psoriatic arthritis, ankylosing spondylitis, other spondyloarthropathies, and systemic lupus erythematosus, or other connective tissue diseases. This method has been shown to have greater than 90% positive predictive value (PPV) for identifying patients with a rheumatologist-reported diagnosis of RA [10]. A group of hospitalised patients free of rheumatic disease throughout the entire study period was matched to patients with RA for age, sex, and index year at a ratio of up to 3:1 and served as the control group. Baseline was the index date representing the date of the first hospitalisation with RA for patients with RA, and for controls, the closest date of admission in the same year. Incident dementia was defined as a person with a first dementia diagnosis in the HMDS after the index date using established relevant ICD, 9th Revision, Clinical Modification or ICD-10-AM codes for AD, vascular dementia (VD), and nonspecific dementia (NSD) (Supplementary Table S1) [11]. A single hospitalisation with dementia has a PPV of 80.4% to identify AD cases with high specificity (99.1%) and moderate sensitivity (79.3%) [12]. Also, dementia as defined in the Charlson comorbidity index (CCI) by the same ICD codes has over 80% probability in identifying true dementia cases and performed as well as the Diagnostic and Statistical Manual of Mental Disorders, Fourth Edition criteria in a retrospective population-based cohort of patients with RA and matched non-RA controls [13]. Patients with dementia diagnosed before the RA index date were excluded from the analysis. Data on comorbidities (smoking, hypertension, type 2 diabetes mellitus, dyslipidaemia, and obesity) were extracted using the relevant ICD 9th and 10th codes (Supplementary Table S1). This study, using deidentified health data, was approved by the Western Australia Health Research Ethics Committee (HREC 2016.24, renewed in June 2021).

### Statistics

Results are presented as median with IQRs, frequencies and proportions, and odds ratios (ORs) or rates with 95% CIs. Patients with RA and controls were followed until the time of death or the end of the study period (January 1, 2015). Incidence rates (IRs) and mortality rates (MRs) were estimated per 1000 person-years (PYs), and the accrued modified CCI (m-CCI) score (ie, excluding rheumatic diseases and dementia) was estimated for the postindex period. Differences between groups were analysed with Mann-Whitney *U* test and the chi-square test with Fisher’s exact test for small numbers. Survival data were described with Kaplan-Meier estimates with log-rank testing for subgroup comparisons. All analysis conducted for the study was done using International Business Machines Corporation (IBM) Statistical Package for the Social Sciences (SPSS) (v29.0) and OpenEpi: https://www.openepi.com/, and two-tailed *P* values <.05 indicated statistical significance.

## RESULTS

**Figure 1:**
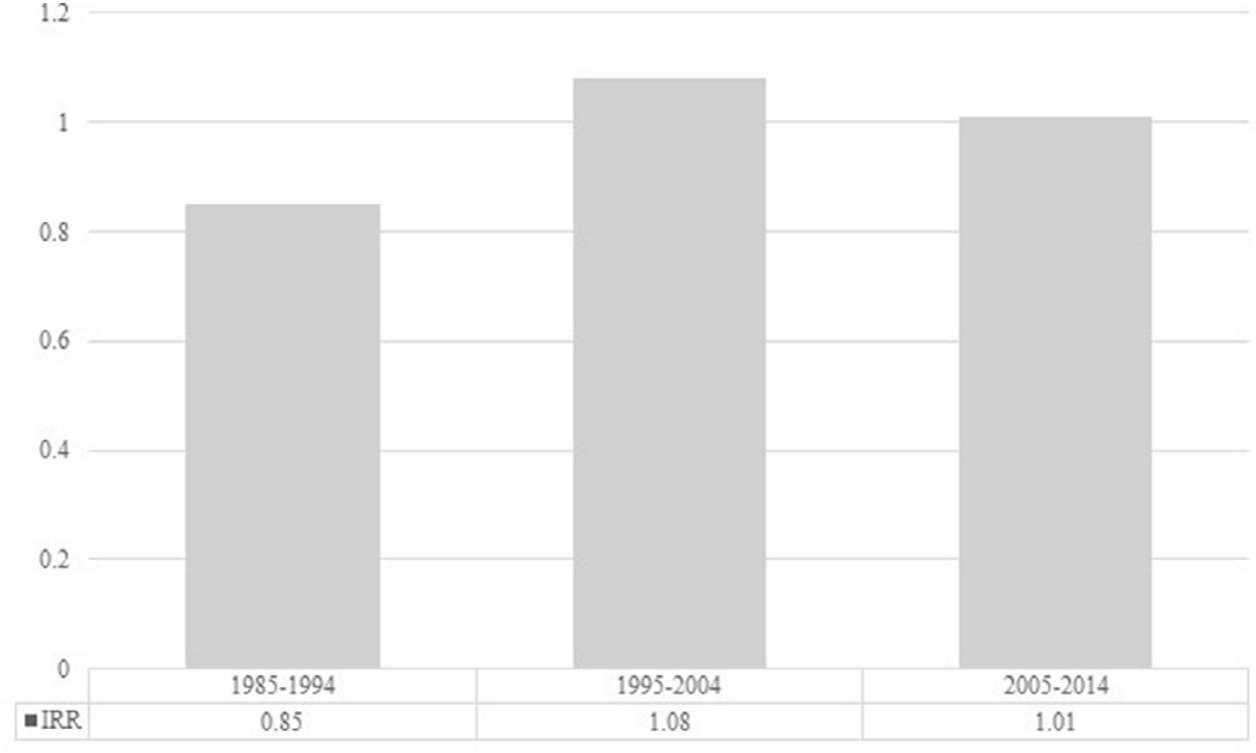
Incidence rate ratio (IRR) for new-onset dementia by study decade for patients with rheumatoid arthritis (RA) and controls.

Patients with RA (n = 14041, index age 64 years, 67.2 % female) and controls (n = 32031, index age 64 years, 65.6 % female) had comparable baseline demographics (Table 1), with the large majority of patients (98.5% and 98.4%) aged 50 and above at baseline. New-onset dementia was diagnosed in 1463 patients with RA (10.4%) and 3710 controls (11 %) during a mean follow-up of 9.6 years (*P* = .07). The overall IR for dementia was 12.07 (95% CI: 11.15-12.71) in patients with RA and 11.59 (95% CI: 11.22-11.97) in controls with an IR ratio (IRR) of 1.04 (95% CI: 0.98-1.11, *P* = .21). Despite a right-skewed distribution in the proportion of cases over time, the IRR did not differ significantly over time (Fig 1). In a time-dependent analysis accounting for the competing risk of death, the time from baseline to dementia diagnosis was not significantly different (67 months; 95% CI: 61.2-72.4 vs 60 months; 95% CI: 55.4-64.6; *P* = .34; (Supplementary Fig S1). Median age at dementia diagnosis (83 vs 84 years) was marginally lower in patients with RA, and there was a female preponderance among dementia patients in both groups (Table 1).

**Table 1:** Descriptives and characteristics of patients with rheumatoid arthritis and controls with incident dementia

	All patients with RA (n = 14,041)	All controls (n = 33,785)	*P* value
Index age	64 (51-75)	65 (51-76)	.01
Total person-years	121,151	320,135	
Nr with incident dementia (%)	1463 (10.4)	3710 (11)	.07
Dementia incidence rate	12.13 (11.15-12.71)	11.59 (11.22-11.97)	.21
Characteristics for patients with dementia			
Index age	76 (69-82)	77 (71-83)	.01
Age at dementia diagnosis	83 (78-87)	84 (79-88)	.14
Time since index (mo)	71 (21-136)	66 (10-148)	.02
Females	1076 (73.5)	2662 (71.8)	.21
Indigenous	20 (1.4)	56 (1.5)	.79
Diabetes mellitus	118 (9.0)	254 (7.9)	.23
Smoking	533 (36.4)	1019 (27.5)	.01
Hypertension	676 (46.2)	1444 (38.9)	.01
Dyslipidaemia	174 (11.9)	372 (10.0)	.05
Obesity	175 (12)	158 (4.3)	.01
Depression	219 (15.0)	369 (9.9)	.01
Anxiety	51 (3.5)	111 (3.0)	.37
m-CCI ≥ 3	1011 (69.1)	2653 (71.6)	.11

m-CCI, modified Charlson comorbidity index; RA, rheumatoid arthritis.

Figures indicate the number of patients (%), incidence rate per 1000 person-years and median time with IQR.

The proportion of patients with hypertension, smoking history, obesity, dyslipidaemia, and depression was higher in the patients with RA than in controls with dementia, but baseline multimorbidity (m-CCI ≥ 3) was similar (Table 1). In multivariate logistic regression analysis, female sex, increasing index age, earlier calendar index year, smoking, hypertension, dyslipidaemia, and a history of depression independently increased the hazard for dementia to a similar extent in both groups. RA was not an independent risk factor for incident dementia, while diabetes mellitus was an additional hazard in controls only (Supplementary Table S2).

Dementia was more frequently classified as NSD in patients with RA (62.7% vs 54.9%, OR = 1.378, 95% CI: 1.217-1.560, *P* < .001) and less frequently as AD (8.5% vs 13.4%, OR = 0.597, 95% CI: 0.484-0.733, *P* < .001) with no difference noted for VD. Overall, 2 or more subtypes of dementia were recorded in 24.1% of patients with RA and 27.9% of controls (OR 0.85, 95% CI: 0.736-0.971, *P* = .017). Since 2000, the proportion of patients classified to have VD decreased (by 6.9% in RA vs 5.5% in controls), whereas the proportion diagnosed with AD increased by 8% in both groups, albeit still lower in patients with RA (Supplementary Fig S2).

Following a dementia diagnosis, readmissions for any cause occurred at a higher rate in patients with RA than in controls (1508 vs 1035 per 1000 PY, *P* < .01) including higher readmission rates for dementia (272 vs 241 per 1000 PY, *P* < .01), stroke, myocardial infarction, fragility fractures, and pneumonia (Table 2) and also a higher rate of emergency department visits (534 vs 405 per 1000 PY, *P* < .01).

**Table 2:** Outcomes for patients with rheumatoid arthritis and controls with dementia

	Dementia group		
	RA (n = 1463)	Controls (n = 3710)	*P* value
Total person-years	13,723	36,534	
Morbidity			
Nr of readmissions (any)	20,695	37,805	
Overall readmission rate	1508.8 (1487.4-1528.5)	1035.2 (1024.3-1046.8)	<.01
Dementia readmission rate	272.1 (263.6-281.2)	241 (236-264)	<.01
RA readmission rate	232.4 (224.4-240.6)	-	
Emergency visits	7327	14,800	
ED visit rate	533.9 (521.8-546.3)	405.1 (398.6-411.7)	<.01
m-CCI accrual	2 (1-3)	2 (1-4)	.12
Pneumonia	138 (9.4)	272 (7.3)	.014
Cancer (any)	353 (24.1)	1139 (30.7)	<.01
Stroke	552 (37.7)	1169 (31.5)	<.01
MI	361 (24.7)	552 (14.9)	<.01
Fracture	711 (48.6)	1542 (41.6)	<.01
Fragility fracture	112 (7.7)	114 (3.1)	<.01
Mortality			
Nonsurvivors	1260	3304	
Overall mortality rate	91.8 (86-8-97.0)	90.4 (87.4-93.6)	.64
Mortality rate for diagnosis <2000	150.1 (136.2-165.4)	156.2 (147.4-165.4)	.47
Mortality rate for diagnosis ≥2000	76.9 (71.8-82.3)	73.3 (70.2-79.5)	.24
Postdementia diagnosis survival	2 (1.76 -2.14)	1.8 (1.67-1.95)	.01
1 y survival	63.7 (CI)	61.4 (CI)	
5 y survival	21.7 (CI)	19.2 (CI)	

ED, emergency department; m-CCI, modified Charlson comorbidity index; MI, myocardial infarction; RA, rheumatoid arthritis.

Figures are numbers (%), median (IQR) and rates per 1000-person-years with 95% CIs.

**Figure 2:**
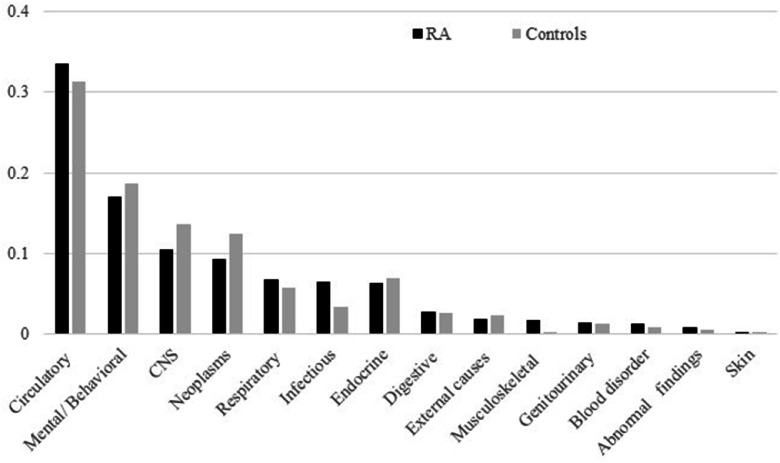
Primary causes of death by main disease category in patients with rheumatoid arthritis (RA) and controls with dementia. CNS, central nervous system.

The overall MR in patients with RA with dementia was 91.8 (95% CI: 86.8-97.0), similar to the MR of 90.4 (87.4-93.6) in controls with dementia (*P* = .64), while pre- and post-2000 MR were also comparable for both groups (Table 2). The main causes of death in all patients with dementia were cardiovascular (31.9%), mental/central nervous system (CNS) disorders (30.8%), and malignancy (11.5%) with more infectious deaths (6.5% vs 3.3%, *P* < .01), and fewer CNS (19.7% vs 26.1%, *P* < .01) and cancer deaths (9.27% vs 12.4% *P* < .01) recorded for patients with RA (Fig 2).

## DISCUSSION

This long-term population-based study showed that dementia incidence was not significantly different among hospitalised patients with RA and matched controls. Although patients with RA suffered more complications following dementia diagnosis due to a higher frequency of pneumonia, fractures, and cardiovascular disease (CVD), MRs were similar.

The prevalence of dementia in both groups (10.4%-11%) falls within the estimated prevalence in Australia of 9% in the 65+ age group and 30% in 85+ individuals [6]. Using multiple data sources, the first study of dementia incidence in Australia recently estimated the IR at 12.5 cases per 1000 persons over 65 years of age [14], which is in line with the dementia incidence in our RA cohort (12.1/1000 PY). These rates are comparable to the 13-20/1000 PY incidence based on the US Medicare claims database and the population-wide dementia incidence (7.2-18.1/1000 PY) in Canada during overlapping periods [12,15]. Together with similarities in sex distribution and age at onset of dementia, these findings indicate that our results are a reliable reflection of wider population data.

The potential impact of RA on dementia was recently reviewed with conflicting results [16]. Of the 10 studies including a control group, the risk of physician-diagnosed dementia in RA was increased for all types of dementia (hazard ratio [HR]: 1.14) in 2 studies, although no longer significant when restricted to patients aged >65 [17,18]. One study reported an increased risk for AD (HR: 1.37) in patients with RA, but only before 2000 [13] and 1 reported an increased risk for VD (HR: 1.16) and reduced risk for AD (HR: 0.89) in patients with RA [19]. The 6 remaining studies using an identical ICD coding-based approach found no increased dementia risk for patients with RA (HR: 0.91-0.96) [[20], [21], [22], [23], [24]]. Our data confirm that the underlying burden of inflammation in patients with RA does not impact the risk of dementia to any significant extent. Biological treatment has been associated with a lower rate of dementia in patients with RA in selected cohorts [15,25,26], and our study period captured the time period when methotrexate (early 1990s) and biological disease-modifying antirheumatic drugs (DMARDs) (early 2000s) were introduced. Our data indicate that the increased availability of more efficacious drugs for RA has not had a measurable early impact on dementia development over a 15-year period, but we cannot exclude that more prolonged periods of use and the introduction of a broader range of biological/targeted (b/t)DMARDs may demonstrate a more pronounced effect in patients with RA in the future.

We confirm the well-established risk factors for dementia, such as age, traditional cardiovascular risk factors, and depression, in both patients with RA and controls. This means efforts to prevent dementia will require a similar approach in both groups, highlighting the importance of cardiovascular risk management in patients with RA [[27], [28], [29]] as biological treatment has been associated with lower cardiovascular risk and a lower rate of dementia in patients with RA [15,25,26]. We also found a slight reduction in the odds for dementia with more recent calendar years, which fits with a small decline in dementia incidence in the United States and stabilisation of dementia occurrence in Europe [30,31].

Distinguishing between the main subtypes of dementia helps guide management decisions. Based on physician-reported diagnoses, which consider the underlying complex of clinical, neuropsychological, and imaging investigations, we found that the prevalence of VD did not differ between groups, whereas AD prevalence was significantly lower and NSD higher in patients with RA. Although our data support other evidence for a lower AD incidence in RA than in controls, this association is not straightforward [19,32]. A Mendelian study found that the genetic susceptibility for RA was associated with a lower risk for AD [33], but a genome-wide analysis found no such protective effect [34]. Although treatment with b/tDMARDs or synthetic DMARDs is associated with lower AD risk in some studies [15,35,36], the proportion of patients diagnosed with AD in our cohort increased by 8% in both groups since 2000 following the introduction of bDMARD. The lack of specific medication details and additional confounders, however, precludes a more definitive conclusion [29]. We did observe a lower rate of mixed-type dementia in patients with RA, but comparative studies are lacking and discussion about the true or artificial nature of mixed-type dementia is ongoing [37].

Patients with RA and dementia had significantly higher rates of overall and dementia-related hospital admissions and emergency department visits, with more patients with RA and dementia admitted for stroke, myocardial infarction, and (fragility) fracture. This indicates an added burden from extra-articular complications in patients with RA and dementia. There are little data on the effect of dementia on RA activity, but the readmission rate for RA of once per 5 years was largely in line with US data for patients >65 years [38], suggesting dementia did not have a large impact on RA flares requiring admission.

Dementia is ultimately a fatal condition, as illustrated by the median survival of just 2 years and the 20% survival rates 5 years after dementia diagnosis. Similar to the incidence data, the MR remained similar for both groups in the post-2000 era, where more efficacious drug therapy was available. The cause of death data indicated that mental disorder/CNS disease and CVD together made up two-thirds of all causes of death, illustrating the close association between CVD and dementia, compounded by the advanced age at dementia diagnosis and not influenced by the presence of RA.

The limitations of the study should be kept in mind. They include the possibility of underestimating the true rate of dementia, although ICD-based case identification has over 80% PPV with high specificity [12]. Despite an average all-cause admission rate of once every 4 years, we may have missed dementia in patients with RA who were never hospitalised over the long observation period [39]. The use of a physician-based diagnosis of dementia provides the best epidemiological standard [40], as it implies that the composite of memory loss, problem-solving, and communication difficulties was sufficient to warrant a dementia diagnosis. Also, there was 96% concordance between our diagnostic criteria and the m-CCI algorithm for dementia (data not shown). We lacked data on the speed or extent of cognitive decline, and with 10 years of follow-up of patients with a median age of 67 years, we may not have fully captured the age-dependent incidence of dementia. However, this limitation applies equally to patients with RA and controls. Our administrative dataset lacks clinical detail on RA disease features such as disease activity, autoantibody profiles, and medication, which precluded an analysis of these potential risk factors for dementia.

The strength of this study is the population-wide and long-term study of a large RA cohort and a control group to investigate an aspect of RA, for which there are limited data.

### Conclusions

Dementia incidence in patients with RA remained similar to that of controls over a 30-year period. Although patients with RA were less likely to be classified as AD and required more subsequent hospital care, MRs over time did not differ. Taken together, these long-term data show that RA did not significantly impact the development or outcome of dementia.

## Appendix Supplementary materials

Image, application 1

## Footnotes

Supplementary material associated with this article can be found in the online version at doi:10.1016/j.ero.2026.03.007.
