# Supplementary material for: Temporal incidence and impact of dementia in rheumatoid arthritis: a cohort study
Source: EULAR Rheumatol Open. 2026 Apr 8;2(2):100159. doi: 10.1016/j.ero.2026.03.007 (PMC13425154; doi:10.1016/j.ero.2026.03.007)
Supplement: Supplementary file 1 [file mmc1.docx]

**Supplemental info for manuscript “ Temporal incidence and impact of dementia in Rheumatoid Arthritis – a cohort study”**

Supplementary Table S1 International Classification of Disease (ICD) codes applied to define conditions.

|  | ICD9-CM | ICD10-AM |
| --- | --- | --- |
| Rheumatoid Arthritis | 714.x | M05.x-M06.x |
| Dementia (any type) | 331.0, 290.4x, 290.0-290.3, 294.1, 294.2, 331.1, 331.2, 331.8, 331.9 | F00.x- F01.x, G30.x, F02.8, F03.0 , F05.1, G31.0, G31.1, G31.3, G31.8, G31.9 |
| Alzheimer’s Dementia | 331.0 | F00.x, G30.x |
| Vascular Dementia | 290.4x | F01.x |
| Non-Specific Dementia | 290.0-290.3, 294.1, 294.2, 331.1, 331.2, 331.8, 331.9 | F02.8, F03.0 , F05.1, G31.0, G31.1, G31.3, G31.8, G31.9 |
| Depression | 296.2-296.4, | F31.3-5, F32.x,F33.x |
| Anxiety | 293.84, 300.x | F06.4, F41.x |
| Smoking (ever) | 305.1, V15.82 | F17, Z72.0, Z86.43 |
| Arterial hypertension | 401-405 | I10-I13, I15 |
| Hyperlipemia | 272.x | E78.x |
| Diabetes Mellitus | 250 | E10-E14 |
| Obesity | 278.0, 278.1 | E65.x, E66.x |
| Charlson Comorbidity Index | Quan et al  *Med Care* 2005;43(11):1130-9 | Quan et al  *Med Care* 2005;43(11):1130-9 |

Supplementary Table S1 Hazard ratios (HR) for clinical factors independently associated with incident dementia in patients with Rheumatoid Arthritis (RA) and controls.

|  | **RA** | | | **Controls** | | |
| --- | --- | --- | --- | --- | --- | --- |
|  | HR | CI | p- value | HR | CI | p- value |
| Index Age | 1.079 | 1.073- 1.085 | <.001 | 1.085 | 1.081-1.089 | <.001 |
| Index Year | 0.956 | 0.948- 0.964 | <.001 | 0.95 | 0.945- 0.954 | <.001 |
| Female | 1.352 | 1.185- 1.542 | <.001 | 1.317 | 1.213- 1.43 | <.001 |
| Indigenous Separations(1) | 2.423 | 1.474- 3.982 | <.001 | 1.289 | 0.957- 1.736 | 0.095 |
| Dyslipidaemia | 1.249 | 1.04- 1.499 | 0.017 | 1.457 | 1.285- 1.653 | <.001 |
| Hypertension | 1.184 | 1.05- 1.336 | 0.006 | 1.078 | 0.996- 1.167 | 0.062 |
| Smoking | 1.345 | 1.178- 1.536 | <.001 | 1.175 | 1.075 -1.283 | <.001 |
| Depression Diagnosed(1) | 2.727 | 2.291- 3.246 | <.001 | 3.211 | 2.801- 3.68 | <.001 |
| DM | - | - | - | 1.16 | 1.008- 1.336 | 0.038 |
| Obesity | - | - | - | 0.721 | 0.603- 0.862 | <.001 |

Supplementary Figure S1 Kaplan Meyer survival curve for time from baseline (index date) to dementia diagnosis accounting for competing risk of death in patients with Rheumatoid Arthritis (RA) and controls.


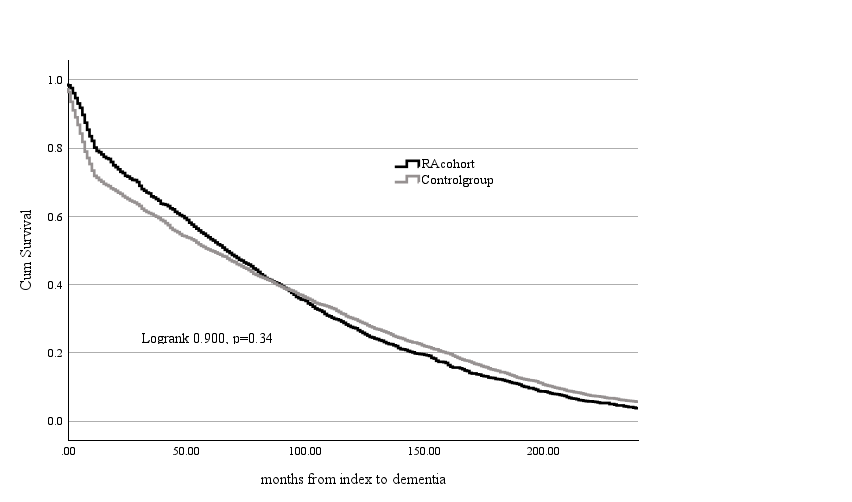


Supplementary Figure S2 Proportion of patients with Rheumatoid Arthritis (RA) and controls diagnosed with various subtypes dementia before

and after the year 2000. AD: Alzheimer’s disease, VD: vascular dementia, NSD : nonspecific dementia
